# Metabolic reprogramming and epigenetic modifications in cancer: from the impacts and mechanisms to the treatment potential

**DOI:** 10.1038/s12276-023-01020-1

**Published:** 2023-07-03

**Authors:** Xuemeng Xu, Qiu Peng, Xianjie Jiang, Shiming Tan, Yiqing Yang, Wenjuan Yang, Yaqian Han, Yuyu Chen, Linda Oyang, Jinguan Lin, Longzheng Xia, Mingjing Peng, Nayiyuan Wu, Yanyan Tang, Jinyun Li, Qianjin Liao, Yujuan Zhou

**Affiliations:** 1 Hunan Key Laboratory of Cancer Metabolism, Hunan Cancer Hospital and the Affiliated Cancer Hospital of Xiangya School of Medicine, Central South University, Changsha, 410013 Hunan China; 2 University of South China, Hengyang, 421001 Hunan China; 3 Hunan Key Laboratory of Translational Radiation Oncology, 283 Tongzipo Road, Changsha, 410013 Hunan China

**Keywords:** Cancer metabolism, Biomarkers

## Abstract

Metabolic reprogramming and epigenetic modifications are hallmarks of cancer cells. In cancer cells, metabolic pathway activity varies during tumorigenesis and cancer progression, indicating regulated metabolic plasticity. Metabolic changes are often closely related to epigenetic changes, such as alterations in the expression or activity of epigenetically modified enzymes, which may exert a direct or an indirect influence on cellular metabolism. Therefore, exploring the mechanisms underlying epigenetic modifications regulating the reprogramming of tumor cell metabolism is important for further understanding tumor pathogenesis. Here, we mainly focus on the latest studies on epigenetic modifications related to cancer cell metabolism regulations, including changes in glucose, lipid and amino acid metabolism in the cancer context, and then emphasize the mechanisms related to tumor cell epigenetic modifications. Specifically, we discuss the role played by DNA methylation, chromatin remodeling, noncoding RNAs and histone lactylation in tumor growth and progression. Finally, we summarize the prospects of potential cancer therapeutic strategies based on metabolic reprogramming and epigenetic changes in tumor cells.

## Cancer: Epigenetic changes alter metabolism in tumor cells

Understanding how epigenetic changes, changes to DNA that alter gene expression without changing the genetic code, alter tumor metabolism may help to identify new cancer treatments. The metabolism of sugars, fats, and amino acids in cancer cells is altered to fuel their unlimited growth, and these alterations are often brought about by epigenetic changes, such as changes in the three-dimensional structure of DNA. Xuemeng Xu at Central South University in Changsha, China, and co-workers review recent research on the types of epigenetic modifications that regulate reprogramming of tumor metabolism. They also report that some metabolites produced by the altered metabolism, such as lactate, are used in epigenetic modification, leading to further changes. Because epigenetic changes, unlike mutations, are reversible, these results indicate some promising pathways for research into cancer treatment.

## Introduction

Cancer is a complicated disease resulting from the proliferation and metastasis of malignant cells. Although some progress has been made in understanding cancer etiology over the years, tumor pathogenesis has not been clarified^1^. Cell metabolism maintains the normal life activities in the body, providing energy and growth products needed for life activities and maintaining the balance in cellular redox. In the process of tumorigenesis and tumor development, cell metabolism is changed, meeting the energy and biosynthetic needs of the uncontrolled proliferation of cancer cells^2,3^. For example, the oxygenated glycolysis rate (Warburg effect) is increased in tumors^4^. Although different cancer types exhibit broad heterogeneity and genetic diversity, some metabolic changes are common to cancer cells, and these types of metabolic reprogramming are thought to be hallmarks of cancers^5,6^. Our understanding of cancer cell metabolic reprogramming is far from sufficient. Therefore, it is important to elucidate the specific molecular mechanisms underlying metabolic reprogramming. Epigenetics was first proposed by Conrad Waddington, a British developmental biologist, in the 1950s. He defined epigenetics as “a branch of biology that studies the relationship between genes and their products, thus forming phenotypes”^7^. On the basis of this definition, epigenetics originally referred to all the molecular pathways in which the expression of a genotype is regulated to generate a specific phenotype. With the advent of genetics, the definition of epigenetics has changed, and now, it mainly refers to both mitotically and meiotically stable and heritable variations in gene expression that do not involve alteration to the DNA sequence^8^. Epigenetic modifications mainly include DNA methylation, posttranslational modifications of histone proteins, chromatin remodeling and noncoding RNA-induced regulation. Epigenetic modifications impact physical and psychological development^9,10^, and abnormal epigenetic modification has been shown to be involved in the development and progression of various diseases^11^. Numerous studies have reported that aberrant regulation of epigenetic modifications can induce tumorigenesis and tumor progression through metabolic reprogramming of cancer cells^12^.

**Fig. 1:**
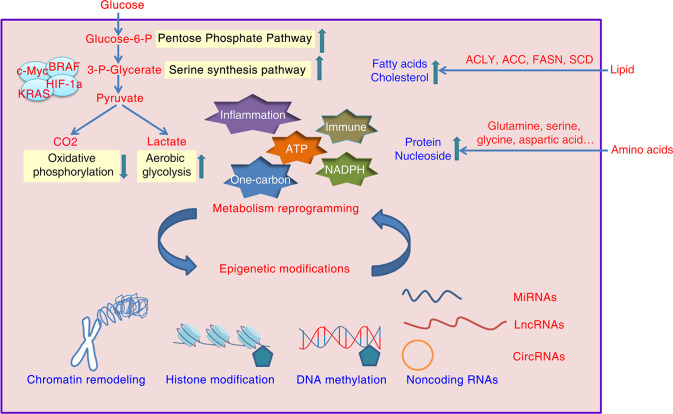
Metabolic reprogramming pathways and epigenetic modification marks interact in cancer. In cancer cells, metabolic pathways are altered during tumorigenesis and development, exhibiting regulated metabolic plasticity. Cancer metabolic reprogramming involves mainly a shift from oxidative phosphorylation to aerobic glycolysis, increased pentose phosphate pathway and serine synthesis pathway activation, and enhanced lipid and amino acid metabolism in cancer cells, providing essential raw materials and energy support for tumor growth, participating in the tumor immune response, and maintaining redox homeostasis in the tumor microenvironment. Metabolic changes are often closely related to epigenetic changes. The mechanisms of epigenetic modification mainly include DNA methylation, histone modification, chromatin remodeling and noncoding RNA functions. Epigenetic modification marks and metabolic reprogramming pathways interact to play an essential role in tumorigenesis and development.

In recent years, with the implementation of cancer cell genome sequencing projects and advances in cancer research, the role of epigenetics in tumorigenesis has been widely accepted^12–16^. An increasing number of studies have shown that cancers are the results of multiple factors, such as the environment and genetics^17^. Epigenetic modification regulation of metabolic reprogramming in cancer cells provides a reasonable explanation for gene-related phenotype changes in the absence of changes to the DNA sequence during tumorigenesis. The effects of epigenetic modification and metabolic reprogramming on cancer cells are not independent of each other. Studies have revealed that metabolic reprogramming in cancer cells remodels tumor epigenetic modifications^18,19^. In this review, we focus mainly on the regulatory role played by different epigenetic modifications during metabolic reprogramming in cancer cells (Fig. 1). First, we briefly introduce the changes in glucose, lipid, and amino acid metabolism in cancer cells. Then, we discussed the regulatory effects of DNA methylation, histone modification, chromatin remodeling, and noncoding RNA on metabolic reprogramming in tumor cells. Finally, we discuss the treatment of tumors based on metabolic reprogramming and epigenetic modification.

## Metabolic reprogramming in cancer

### Glucose metabolism

Glucose is the main source of energy for cell proliferation. Glucose metabolism includes the glycolytic pathway, pentose phosphate pathway (PPP), and serine synthesis pathway (SSP) in the cytoplasm, as well as oxidative phosphorylation, that is, the tricarboxylic acid cycle (TCA), in mitochondria. Glycolysis is the common process through which glucose is metabolized. Pyruvate, a metabolic product of glycolysis, can be converted to lactate and secreted out of a cell, or it can enter the mitochondria for consumption in the TCA. The intermediate products from glycolysis can enter the PPP pathway and SSP pathway. Warburg first discovered that cancer cells produce lactic acid through glycolysis to provide energy even when the oxygen level is sufficient for oxidative phosphorylation; this metabolic feature of cancer cells is called the “Warburg effect” or “aerobic glycolysis”^20^. This change in the glucose metabolism pathway is the most common and representative phenotype of the glucose metabolism change in cancer cells and constitutes the main driving factor in cancer progression^21^. Research has shown that abnormal metabolism in cancer cells is related to abnormal expression of the proto-oncogenes cMyc, KRAS, and BRAF. cMyc regulates the expression of most glycolytic enzymes, thereby transforming the metabolism of cancer cells from oxidative phosphorylation to glycolysis, and abnormal expression of cMyc drives serine synthesis pathway activation in tumors under nutrient-deficient conditions^22^. Oncogene KRAS mutations alter cancer cell metabolism by inducing upregulated transcription of glucose transporters and glycolytic enzymes. Hypoxia-inducible factor (HIF) is the main regulatory factor in glucose metabolism in the hypoxic microenvironment of tumor cells. HIF-1a enhances hypoxia-related glycolysis in cancer cells by activating the expression of multiple glycolytic enzymes, which is crucial for the growth and proliferation of cancer cells under hypoxic conditions^23–25^. In addition, the stability and activity of HIFs are affected by the TCA cycle, electron transport chain components, mitochondrial respiration, and mitochondrion-associated proteins. Activation of HIF is associated with mitochondrial dysfunction, including the oxidative capacity in mitochondria, biogenesis, apoptosis, and autophagy^26^. Glycolysis is an important carbon process in cancer cell metabolism, providing nutrients for the unlimited proliferation of cancer cells. The intermediates of glycolysis are consumed to promote the biosynthesis of biomolecules, such as nucleotides, amino acids, and fats^27^. Glycolysis leads to high levels of accumulated lactic acid in the tumor microenvironment. Lactate is a signaling molecule in cancer, regulating inflammation and immune responses in tumors^28^. In addition, enhanced aerobic glycolysis in cancer cells promotes angiogenesis^29^. Abnormal glucose metabolism as a hallmark of cancer has been used in clinical diagnostics and treatment. For example, fluorodeoxyglucose positron emission tomography (18F-FDG-PET) has been widely used in tumor diagnosis, exploiting the difference between glucose metabolism in tumors and normal tissues^30,31^. Specifically, glucose metabolism provides preferential targets for cancer therapy. Tumor growth can be limited by inhibiting glycolysis; for example, the ruthenium-based anticancer compound BOLD-100 targets glycolysis and renders cells vulnerable to glucose-deficient metabolism^32^.

### Lipid metabolism

Lipids are biological macromolecules that are the bases of biological membrane structure and function as signaling molecules and energy sources^33^. During the development of cancer, fat metabolism dramatically changes^34^. Some key enzymes such as ATP-citrate lyase (ACLY), acetyl-CoA carboxylase (ACC), and fatty acid synthesis enzyme (FASN) involved in fatty acid metabolism in cancer cells are upregulated in certain cancers, including liver cancer, and are related to clinical prognosis^35–37^. Under hypoxic conditions, the fatty acid synthesis rate in cancer cells increases. Fatty acid intake and metabolism promote cancer progression. Studies have shown that rapidly proliferating cholangiocarcinoma cells depend on the uptake of lipids and lipoproteins to promote FA catabolism^38^. Recently, fatty acid metabolism pathways as potential antitumor targets have attracted increasing attention^39^.

The involvement of abnormal cholesterol metabolism is also important in cancer development. A recent study found that dysregulation of cholesterol homeostasis leads to resistance to iron, dyslipidemia, or hypercholesterolemia, thereby increasing tumorigenesis and metastasis by resisting ferroptosis^40^. Cholesterol participates in the inflammatory response by regulating various immune cell functions. Thus, controlling cholesterol metabolism may increase the cancer immune response^41^. For example, regulatory T cells (Treg cells) drive immunosuppression in the tumor microenvironment. Lipid synthesis and metabolic signals in Treg cells rely on steroid regulatory element-binding proteins (SREBPs) to induce potent antitumor immune responses without increasing toxicity related to autoimmunity. Recent research has shown that the activity of SREBPs is upregulated in Treg cells within tumors. SREBP lysis activation protein (SCAP) is required for SREBP activity. SCAP-deficient Treg cells suppressed tumor proliferation and promoted the initiation of immunotherapy by targeting the immune checkpoint protein PD-1. The lipid signaling pathway enhances the specialized function of Treg cells in tumors^42^.

### Amino acid metabolism

In addition to increased aerobic glycolysis and fatty acid synthesis, cancer cells show an increased demand for amino acids. An adequate provision of amino acids is vital for cancer cells to sustain their proliferative dynamics. In addition to a substrate for protein synthesis, amino acids are important for energy production, driving nucleoside synthesis, and maintaining cellular redox balance^43^.

Although the energy needed for cancer metabolism is derived mainly from aerobic glycolysis, amino acids such as glutamine, serine, and glycine play key roles in cancer metabolism^44,45^. Serine, a major source of one-carbon units, plays an essential role in tumor development. The rapid proliferation of tumor cells depends on the supply of extracellular serine. Baksh et al.^46^ found that epidermal stem cells (EpdSCs) rely on extracellular serine for metabolic reprogramming that inhibits their differentiation, thereby boosting the proliferation of cancer-causing cells. Restricting serine intake can limit tumor growth by altering sphingolipid diversity^47^. Moreover, inhibition of the serine synthesis pathway and reduced dietary serine and glycine levels showed clear positive effects on cancer treatment^48^. Nucleotide synthesis also depends on amino acids. Glycine, glutamine, and aspartate provide carbon and nitrogen for the biosynthesis of purines. Glycine, serine, and methionine provide carbon units through the methionine–folate cycle. Amino acid metabolism is also important in maintaining redox homeostasis. Glutathione is an important intracellular reactive oxygen scavenger. In tumors, high reactive oxygen species (ROS) levels disrupt the redox homeostasis of the tumor microenvironment. Tumor cells show an increase NADPH levels, mediated through various mechanisms, enabling their adaptation to high ROS levels, including activation of AMPK and the pentose phosphate pathway (PPP) and reduced glutamine levels and folate pathway activation^49^. Glutamine promotes NADPH production through malic enzyme 1 (ME1), and malate from glutamine in mitochondria is shuttled to the cytoplasm to generate NADPH^50^.

Other substances, such as nucleotides, are subjected to metabolic reprogramming in cancer cells. Metabolic such as 6-phosphate-glucose and one-carbon metabolism link nucleotide metabolism with glucose metabolism and amino acid metabolism. In summary, metabolic reprogramming regulates tumor progression and significantly affects many biological properties of cancer cells, promoting their proliferation, growth, invasion, and distant metastasis.

## Epigenetic modifications regulate metabolic reprogramming in cancer cells

Four main types of epigenetic modifications have been reported: DNA methylation, histone modification, chromatin remodeling, and noncoding RNA-induced modification. DNA methylation maintains a dynamic balance in the body to maintain normal physiological functions. Most histone modification targets are conserved lysine residues in histone tails. The N-terminus of histones can be posttranslationally modified by methylation, acetylation, lactylation, glycosylation, propionylation, or butyrylation. These modifications change DNA–histone interactions, regulating the loosening and condensation of chromatin, thereby activating and repressing transcription, respectively, to alter gene expression. Chromatin remodeling refers to changes in chromatin structure without changes in the covalent modifications of DNA and histones; that is, nucleosome disassembly (separation of DNA and histones), nucleotide translocation, DNA–histone affinity and chromatin three-dimensional structure are unchanged. Noncoding RNAs (NcRNAs), mainly composed of microRNAs (miRNAs), long noncoding RNAs (lncRNAs), and circular RNAs (circRNAs), have been gradually shown in recent years to be crucial to various biological processes.

Epigenetic modifications may influence gene expression and do not alter the DNA sequence. Notably, epigenetic modifications determine the phenotypes of cells and even individuals in a heritable fashion, but the metabolic reprogramming they induce does not involve DNA sequence changes such as those induced by genetic mechanisms. The impact of epigenetic regulation on metabolic reprogramming in cancer cells cannot be ignored. An in-depth understanding of the mechanisms underlying epigenetic regulation of metabolic reprogramming in cancer has far-reaching scientific significance.

### DNA methylation regulates metabolic reprogramming in cancer cells

DNA methylation, referring mainly to the methylation of the 5-carbon cytosine residue (5mC) in a cytosine-guanine (CpG) dinucleotide, was one of the first discovered and most extensively studied epigenetic modifications. Methylation in the body maintains a dynamic balance to maintain normal physiological functions. Abnormal methylation of DNA in cells may contribute to the development of tumors and cardiovascular and autoimmune diseases^51–53^. In tumor cells, abnormal methylation leads to the activation of some important proto-oncogenes, leading to tumor suppressor gene silencing, genomic instability, and chromatin changes. These factors promote tumorigenesis by altering cancer metabolic pathways^53^.

**Fig. 2:**
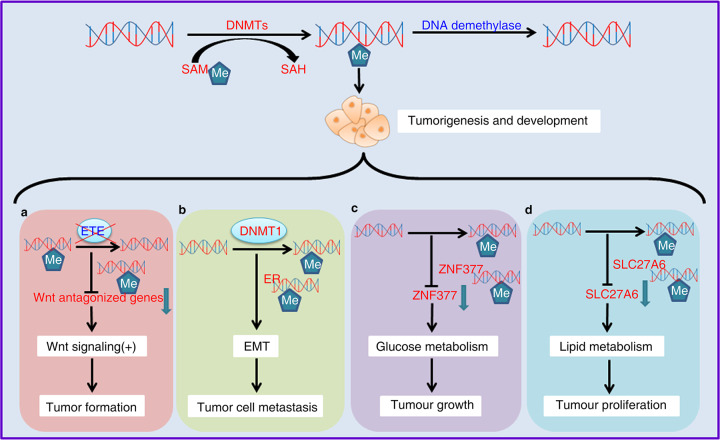
DNA methylation affects tumor initiation and progression. **a** In pre-adenocarcinoma in the lung, DNA hypermethylation caused by functional mutation of TET downregulates the expression of Wnt antagonist genes, triggering abnormal activation of Wnt signaling and accelerating the formation of early tumor lesions. **b** DNMT1-mediated hypermethylation of the estrogen receptor (ER) promoter region inhibits the expression of the ER gene during the epithelial-mesenchymal transition, promoting the EMT, which required for metastasis, thereby promoting the proliferation of cancer stem cells in triple-negative breast cancer (TNBC). **c** Zinc finger ZNF377 negatively regulates the glucose metabolism pathway in tumor cells to exert a significant antitumor effect. **d** SLC27A6 increases the tumor cell metastatic potential by promoting lipid biosynthesis. DNA hypermethylation downregulates the expression of SLC27A6, which promotes the proliferation of nasopharyngeal carcinoma via the regulation of lipid metabolism.

Epigenetic modifications are reversible. The dioxygenase ten-eleven translocation (TET) enzyme promotes DNA demethylation, which is often dysregulated in cancer. In lung adenocarcinoma tissues with functional TET mutations, DNA hypermethylation downregulates the expression of Wnt-antagonized genes, thereby triggering aberrant Wnt signaling activation and accelerating tumor formation (Fig. 2a)^54^. Hypermethylation of the DNA methyltransferase 1 (DNMT1) gene is thought to be a cause of triple-negative breast cancer (TNBC). DNMT1 inhibits the expression of downstream EMT-related target genes through hypermethylation of the estrogen receptor (ER) promoter region, thereby promoting tumor cell metastasis (Fig. 2b)^55^. Brain-expressed X-catenin 1 (BEX1) is highly expressed in hepatoblastoma (HB) and the cancer stem cells of hepatocellular carcinoma (CSC-HCC) patients but expressed at low levels in non-CSC-HCC patients. The differential expression of BEX1 in tumor and normal tissues is regulated by DNMT1, which maintains the self-renewal capacity of the hepatocellular carcinoma CSCs by activating the Wnt/β-catenin signaling pathway. Therefore, BEX1 may be a potential therapeutic target in HB and CSC-HCC. Abnormal glucose metabolism in cancer is linked to DNA methylation. Studies have shown that hyperglycemia levels inhibit DNA 5-hydroxymethylation. The stability of the DNA demethylase TET2 depends on the phosphorylation of AMPK at serine 99. Increased glucose levels hinder the AMPK-mediated phosphorylation of serine 99, causing TET2 instability, which in turn leads to the dysregulation of 5-hydroxymethylcytosine (5HmC), limiting the tumor suppressor function of TET2^56^. The TET3 protein is commonly upregulated in AML patients and human leukemia stem cells (LSCs). Overexpression of TET3 induces the expression of genes related to glucose metabolism and promotes AML progression^57^. A recent study revealed that the zinc finger DHHC-1 (ZDHHC1, also known as ZNF377) negatively regulated the tumor glucose metabolism pathway and pentose phosphate pathway (PPP), thereby playing a significant antitumor role, making it a potential new tumor suppressor (Fig. 2c). ZDHHC1 is silenced in multiple cancers because of promoter methylation. A study showed that the methylation level of the ZDHHC1 promoter was markedly increased in tumor tissue compared with neighboring normal tissue. In ZDHHC1-deficient cells, ZDHHC1 was expressed after demethylation. These results showed that the methylation of the ZDHHC1 promoter silenced its expression in tumors, thereby inhibiting its regulatory metabolic function^58^. In gliomas, the low expression of ankyrin repeat and death domain-containing 1 A (ANKDD1A) was due to the aberrant methylation of a promoter CpG. ANKDD1A directly interacts with FIH1 to inhibit HIF1 transcriptional activity and shortens the half-life of HIF1 by upregulating FIH1 expression, thereby reducing glucose uptake and lactate production, inhibiting autophagy, and inducing the apoptosis of glioblastoma multiforme cells. However, in glioma multiforme, the high level of ANKDD1A methylation changes the metabolism of the cancer cells, thereby inhibiting the antitumor effect of ANKDD1A^59^. DNA methylation can also affect the development of tumors by regulating the lipid metabolism pathway. For example, solute carrier family 27 member 6 (SLC27A6) increased the levels of triglyceride (TG) and total cholesterol (T-CHO) in nasopharyngeal carcinoma (NPC) cells by promoting lipid biosynthesis. Downregulation of SLC27A6 expression by DNA hypermethylation promoted the proliferation but inhibited NPC cell metastasis by regulating lipid metabolism (Fig. 2d)^60^.

### Histone modification regulates metabolic reprogramming in cancer cells

#### Histone methylation

Methylation modulate cell physiology by regulating the state and activity of histones. Histones H3 and H4 are methylated mainly on a lysine or arginine residue. Histone methylation alters cellular metabolic processes by activating or repressing gene expression^61,62^. The expression of various histone methylases and demethylases plays an important role in histone methylation.

Elevated histone methylation activity of the histone H3 lysine 36 (H3K36) methyltransferase NSD3 has been related to squamous cell lung cancer. According to previous studies, NSD3 increased the transformation of tracheobronchial cells and the growth of transplanted human lung squamous cell carcinoma (LUSC) cells by amplifying 8p11-12 in a catalytic activity-dependent manner^63^. Lysine methyltransferase 2D (KMT2D) regulates tumorigenesis through metabolic reprogramming. KMT2D was transcriptionally repressed by DNA methylation in pancreatic tumors. Inhibition of KMT2D promotes the metabolic transition to aerobic glycolysis by regulating glucose transporter 3 (SLC2A3), increasing the aerobic glycolysis rate and changing the lipid composition of pancreatic cancer cells. The mechanisms regulating KMT2D expression and its downstream effects in pancreatic tumorigenesis represent a potential therapeutic approach to pancreatic cancer as an epigenome-regulated metabolic disease^64^. KMT2D is often mutated in pulmonary cancer. Lung-specific KMT2D deletion facilitates pulmonary tumorigenesis by impairing the superenhancer of PER2 and upregulates tumorigenesis, including an increase in the glycolysis rate^65^. In melanoma cells, KMT2D deletion led to a genome-wide reduction in the H3K4me1 mark, a gene-activating factor, and subsequent inhibition of IGFBP5-activated IGF1R-AKT, resulting in increased glycolysis in cancer cells^66^. SETD1A is an H3K4 methyltransferase. In gastric cancer tissues, SETD1A promoted GC cell glycolysis by upregulating HIF1α expression. Hence, SETD1A is thought to be an important regulator of HIF1α-mediated glycolysis^67^.

Histone demethylases participate in metabolic reprogramming in cancer cells. JMJD1A is highly expressed in bladder carcinoma and contributes to the proliferation of bladder cancer cells by increasing cellular glucose metabolism^68^. JMJD1A promotes the transcription of PGK1 by regulating H3K9me2 levels in the PGK1 promoter region and interacting with HIF-1α to regulate the glucose metabolism pathway in bladder cancer cells, thus regulating the progression of bladder carcinoma. The histone demethylase KDM5B also acts as an oncogene in breast cancer cell proliferation and migration^69^. KDM5B promotes breast cancer cell proliferation and migration by reprogramming lipid metabolism. KDM5B knockdown in breast cancer cells activated the AMPK protein, reduced the level of lipid metabolism, and inhibited the epithelial-mesenchymal transition (EMT), and reduced their growth and migration ability, revealing novel theoretical evidence for the clinical regulation of tumor metabolism by targeting related genes.

#### Histone acetylation

Histone acetylases (HATs) and histone deacetylases (HDACs) work together to maintain normal levels of histone acetylation. Imbalances in histone acetylation modifications lead to the development of various tumors. Aberrant binding of HDACs at specific promoter regions suppresses normal functional gene transcription, inducing malignancy, such as promyelocytic leukemia–retinoic acid receptor-α (PML–RARα) fusion proteins that reactivate HDACs and inhibit the expression of genes associated with hematopoietic cell differentiation, leading to the progression of acute promyelocytic leukemia (APL)^70^.

Histone acetylation is closely related to lipid metabolism in tumors. The histone deacetylase HDAC3 interacts with SREBP2, a key regulator of cholesterol synthesis, and HMGCR, a cholesterol synthesis-limiting enzyme. Downregulation of HDAC3 activates cholesterol synthesis in gastric cancer cells, which induces ROS production, thereby inducing oxidative stress and apoptosis. The use of the histone deacetylase inhibitor trichostatin A (TSA) enhanced the acetylation of H3 and inhibited PI3K/AKT pathway activation, thereby inhibiting gastric cancer cell proliferation and inducing apoptosis^71^. P300/CBP regulates the expression of metabolism-related enzymes by regulating the acetylation of histone H3K18/K27. Inhibition of p300/CBP expression significantly prevented liver cancer cell proliferation and metastasis^72^. Histone acetylation also plays roles in tumor vascular mimicry by regulating glucose metabolism reprogramming and thus affects tumorigenesis. Bactericidal/permeability-increasing fold-containing family B member 1 (BPIFB1) is hyperexpressed in nasopharyngeal epithelial cells and is markedly downregulated in nasopharyngeal carcinoma tissues; its expression is related to the prognosis of patients with nasopharyngeal carcinoma. Recent studies have shown that BPIFB1 reduced GLUT1 transcription through the regulation of the JNK/AP1 signaling pathway, leading to altered glycolysis. BPIFB1 reduced the acetylation level of histones, further inhibiting the expression of the angiogenic mimicry-related genes VEGFA, VE-cadherin, and MMP2, ultimately leading to the inhibition of angiogenic mimicry in nasopharyngeal carcinoma^73^. Histone acetylation is involved in regulating amino acid metabolism. Arginine is a semi-essential amino acid. It plays an essential role in cellular physiology and is an epigenetic regulator of cancer cell metabolic reprogramming because it regulates epigenetic modifications. In prostate carcinoma, arginine upregulated the expression of a nuclear oxidative phosphorylation (OXPHOS) gene mediated through histone acetylation. Arginine-activated expression of lysine acetyltransferases (KATs) increased the overall levels of histone acetylation and acetyl-CoA and promoted the retention of transcriptional enhancer factor domain 4 (TEAD4) at the promoter/enhancer regions of the aforementioned OXPHOS gene, thereby upregulating the OXPHOS gene and maintaining mitochondrial OXPHOS function^74^.

#### Histone lactylation

**Fig. 3:**
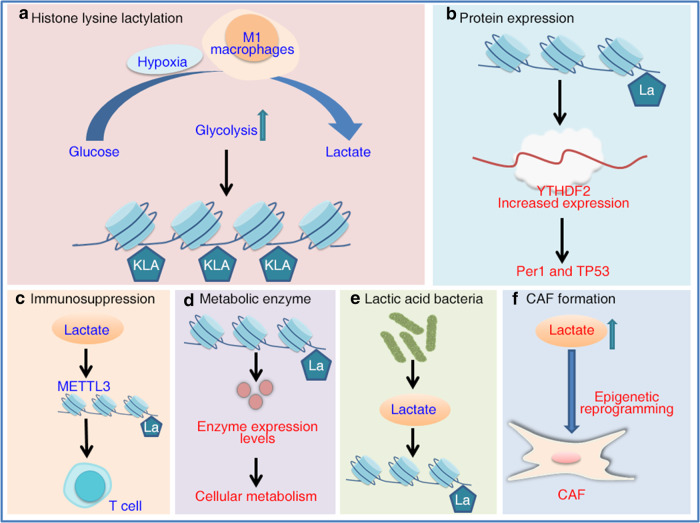
Lactate metabolism and histone lysine lactylation in cancer cells. **a** Lactic acid in the body is derived mainly from the metabolites of glycolysis. Histone lactylation refers mainly to the posttranslational modification of histone lysine residues with lactate as the substrate. Hypoxia and M1 macrophages promote histone lactylation. **b** Histone lactylation promotes the expression of YTHDF2, which promotes the degradation of the mRNA of tumor suppressor genes Per1 and TP53, accelerating ocular melanoma tumorigenesis. **c** The M6A modification of JAK1 mRNA in TIM cells is mediated by the lactylation of METTL3 at K281 and K345 and promotes the immunosuppression of tumor-infiltrating myeloid cells. **d** In non-small cell lung cancer, histone lactylation regulates the expression levels of metabolic enzymes that modulate cancer cell glycolysis. **e** Lactic acid-producing bacteria may promote gastric carcinogenesis by increasing lactate levels and lactylation rates. **f** In pancreatic ductal adenocarcinoma, increased tumor cell-produced lactate flux mediates epigenetic reprogramming to regulate the formation of human pancreatic CAFs.

Historically, lactate was regarded as a metabolic waste product of anaerobic glycolysis in cells. New findings suggest that in addition to being a key metabolite, lactate is a multifunctional biological signaling molecule. Lactate regulates metabolic processes both inside and outside a cell. Additionally, it plays multiple biological roles and is important to cell signaling and immune regulation (Fig. 3)^28,75–78^. Glycolysis produces lactic acid, leading to an acidic tumor microenvironment, which adds complexity to the metabolic heterogeneity of cancer cells.

##### Discovery and mechanism of histone lysine lactylation

In recent years, lactate has been identified as a substrate for the posttranslational modification of lysine residues in histones. This novel epigenetic modification is called histone lysine lactylation (KLA). Zhang et al.^79^ identified 26 and 16 histone KLA sites in human HeLa cells and mouse bone marrow-derived macrophages (BMDMs), respectively. Experiments performed by metabolic labeling with the isotope sodium L-lactate (13C3) and detection by high-performance liquid chromatography (HPLC)–tandem mass spectrometry (MS/MS) indicated that the lactylation of a lysine is based on the lactic acid level. Glucose is the main source of lactate in the body. Glycolysis induces lactate production and regulates histone KLA levels. In addition, both hypoxia and macrophage M1 polarization promote histone lactylation^79^.

##### The role played by KLA in reprogramming cancer cell metabolism

The modification disorder of histone KLA destroys the balance in gene transcription and leads to cancer and other diseases. Higher levels of histone lactylation are associated with poor prognosis in ocular melanoma. Histone lactylation promotes the expression of YTHDF2, which specifically recognizes the m6A modification sites on oncogene Per1 and TP53 RNA and promotes Per1 and TP53 mRNA degradation, thereby accelerating the development of ocular melanoma^80^. Lactylation drives tumor-infiltrating myeloid (TIM) cell immunosuppression. Accumulation of lactate in the tumor microenvironment (TME) significantly induces the expression of methyltransferase-like 3 (METTL3) in infiltrating myeloid cells in colon cancer mediated through H3K18 lactylation, which is essential for the transcription of immunosuppressive genes in TIM cells. This recent finding helps in understanding the molecular mechanisms underlying tumor cell immune escape. Exploring strategies to target TIM cells is important to enhance the T-cell response to tumors^81^. In non-small cell lung cancer, lactic acid regulates cellular metabolism through histone lactylation-mediated expression of oncogenes. Lactic acid inhibits glucose uptake, glycolysis and mitochondrial homeostasis mediated by changes in metabolic enzyme expression levels induced by histone lactylation of gene promoters^82^. In addition, the number of lactic acid-producing bacteria is significantly increased in patients with gastric cancer. Lactic acid-producing bacteria may contribute to the development of gastric cancer by regulating the levels of lactic acid and lactylation modification^83^. In pancreatic ductal adenocarcinoma, increased lactic acid levels have ben associated with epigenome reprogramming during cancer-associated fibroblast formation^84^. In hepatocellular carcinoma, KLA plays an important role in the regulation of cellular metabolism. Researchers have found that KLA regulates amino acid, lipid and nucleic acid metabolism by affecting metabolic enzymes. Moreover, researchers have verified that lactylation promotes the progression of liver cancer^85^.

As a recently discovered posttranslational modification, histone lactylation links cell metabolism to gene regulation, which may indicate its many effects on cancer progression. Studying the functional and regulatory mechanisms of histone lactylation in physiological and pathological processes is crucial and will contribute to an in-depth understanding of the pathogenesis of diseases, including cancers.

#### Other types of histone modifications

**Fig. 4:**
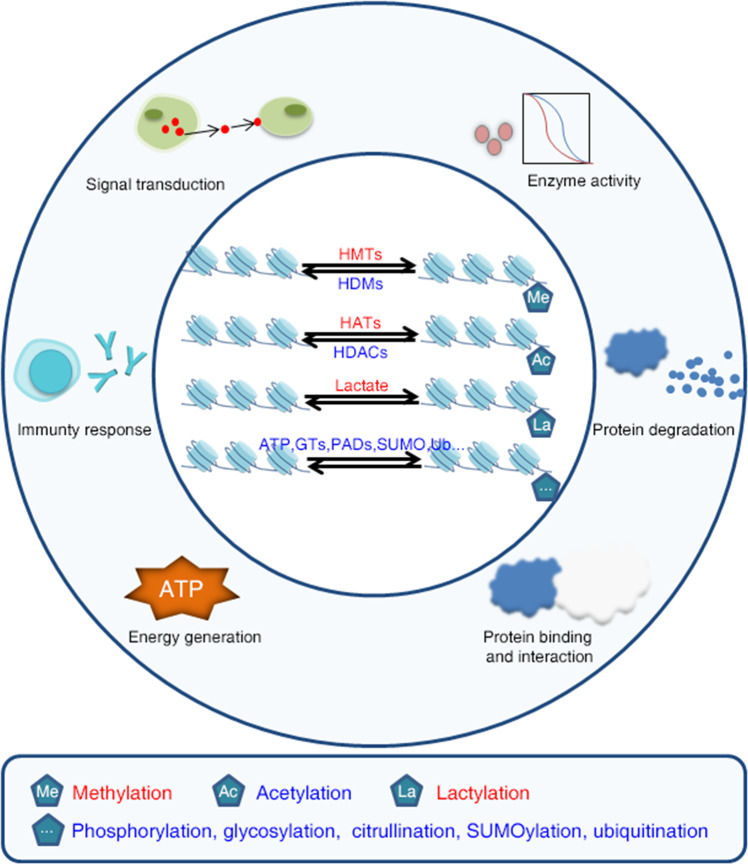
Histone modifications in cancer. Histone tails can be posttranslationally modified by various molecules in the presence of enzymes. Examples include histone methylation, acetylation, phosphorylation, glycosylation, guanylation, succinate, SUMO, and ubiquitination. These histone posttranslational modifications are involved in protein expression and degradation, changes in enzymatic activity, signaling, energy production, the immune response, and various other cancer cell activities.

In addition to methylation, acetylation, and lactylation, the N-terminus and C-terminus of histones can be modified by phosphorylation, glycosylation, citrullination, succinylation, SUMOylation, and ubiquitination (Fig. 4)^18^. All of these posttranscriptional modifications alter the charge and structure of the histone terminus that binds to DNA, thereby altering chromatin status and gene expression^86^. To date, various histone modifications have been identified as important in the metabolic reprogramming of cancer cells^18^.

Histone phosphorylation is not only closely related to ATP production or the energy supply but has been shown to be involved in metabolic reprogramming in cancer cells. For example, Rab26 induces the autophagic degradation of phosphorylated Src by coordinating with autophagy-related 16-like protein 1 (ATG16L1) in breast cancer cells, resulting in the inhibition of cancer cell migration and invasion^87^. Histone citrullination is a posttranscriptional modification that is catalyzed by peptide arginine deiminase (PAD) and is important in physiological and tumor cell regulation. In cancer cells, histone citrullination is related to the formation of neutrophil extracellular traps (NETs), which is conducive to innate immunity and tumor progression^88^. Glycosylation is important in the cellular environment and microenvironment. N-glycosylation and O-glycosylation are the two main glycosylation modifications. A sugar chain is added to the amino group of an asparagine (ASN) residue or the hydroxyl oxygen of a serine/threonine (Ser/Thr) residue. N-glycosylation donors include various branched sugars. O-glycosylation donors include mainly galactose, mannose, fucose, and N-acetylgalactosamine (GalNAc)^89^. Abnormal glycosylation of proteins modulates the acquisition of a malignant phenotype. Epigenetic changes to glycosylation patterns enable cancer cells to evade immune surveillance mechanisms, and targeting glycosylated PD-1 induces effective antitumor immunity^90^. Ubiquitin mediates the proteolysis of cell cycle protein chaperones and kinase inhibitors (CKIs), ensuring precise regulation of the cell cycle. Cell cycle dysregulation caused by inefficient proteolytic regulation leads to an imbalance in cell proliferation and ultimately to tumorigenesis. Therefore, E3 ubiquitin ligases are involved in cell cycle regulation and hold promise as new therapeutic targets in cancer^91^. For example, cullin 3 (CUL3)-mediated beclin 1 (BECN1) degradation inhibits autophagy and promotes cancer progression^92^. UFMylation can maintain the stability of the tumor suppressor p53 by antagonizing its ubiquitination^93^. Taken together, various histone modifications have been shown to exert an essential regulatory effect on metabolic reprogramming in cancer cells, thereby increasing or decreasing tumor progression.

## Chromatin remodeling regulates metabolic reprogramming in cancer cells

Chromatin remodeling is usually catalyzed by a number of ATP-dependent subunit complexes called chromatin complexes; these subunits affect chromatin structure by utilizing energy obtained via ATP hydrolysis and regulate transcription, replication, and DNA damage repair processes, thus playing essential roles in cell growth and tissue development^94^. The dynamically regulated structure of the genome is necessary for timely and proper gene expression in almost all cells. In recent years, complex three-dimensional structure and biochemical properties of proteins have attracted considerable attention. The discovery of chromosome-remodeling complexes has enabled investigations into how alterations to the complicated topology of a protein and the histone landscape mediate crosstalk that leads to oncogenic and carcinogenic effects^95^.

In recent years, chromatin complexes such as SWI/SNF, ISWI, and ANDO80, but especially SWI/SNF complexes, have been found to be linked to a variety of cancers. The SWI/SNF family of chromatin remodeling complexes, also known as BRG1/BRM-related factor (BAF) complexes, are crucial regulators of nucleosome localization. The incidence of mutations in a SWI/SNF subunit is very high, with nearly 25% of cancers having one or more of SWI/SNF gene abnormalities^96^. SWI/SNF gene mutations exert cancer-promoting effects. For example, SWI/SNF complex mutations promote the progression of thyroid tumors^97^. Loss of SNF5, a highly conserved subunit in the SWI/SNF complex, has been found in multiple tumors. Decreased expression of SNF5 promotes bladder cancer progression through the activation of STAT3^98^. The loss of SNF5 expression in melanoma has been associated with a low patient survival rate^99^. ARID1A encodes an SWI/SNF chromatin-remodeling factor. In ovarian clear cell carcinoma cells, ARID1A inactivation increased the accessibility of the GLS1 promoter and upregulated glutaminase (GLS) expression, which depended on glutamine metabolism^100^. In another study, ARID1A deficiency in ovarian cancer cells was shown to impair SWI/SNF recruitment to the SLC7A11 transcriptional start site, thereby reducing cystine uptake and glutathione (GSH) synthesis^101^. Chromatin complexes also participate in the regulation of noncoding RNAs. The SWI/SNF complex regulates the expression of the oncogene miR-222 in lung adenocarcinoma. The SWI/SNF chromosome-remodeling complex has also been associated with lipid metabolism in tumor cells. BRG1 is the catalytic ATPase of the SWI/SNF chromatin-remodeling enzyme. It is a tumor suppressor with obvious effects on certain tumors and is overexpressed in other cancers, thereby increasing or decreasing metabolic pathway activation in cancer cells. For example, BRG1 is overexpressed in breast cancer and promotes tumor cell proliferation by promoting adipogenesis^102^. Knocking out the BRG1 gene reduced the lipid synthesis rate by disrupting enzyme transcription. Targeting BRG1 reduced lipid metabolism, thereby reducing tumor cell proliferation, and therefore, BRG1 is expected to be used in the epigenetic treatment of TNBC. ISWI and INO80 family members show high gene expression and many genetic abnormalities in human cancers^103^. Loss of the ISWI ATPase SmarC5 (SNF2H) inhibited cell proliferation and chromatid cohesion in the AML context^104^. In the melanoma context, INO80 regulated superenhancer (SE)-mediated oncogenic transcription and tumor progression^105^. Decomposition of an r-loop by INO80 facilitated DNA replication and sustained the capacity of cancer cells to proliferate and survive^106^. These studies summarize the link between ATP-dependent chromosome-remodeling complexes and cancer metabolism and demonstrate the mechanisms by which aberrant chromosome-remodeling complex components play roles in tumorigenesis, suggesting possible new ideas for tumor therapy.

## Noncoding RNAs (ncRNAs) regulate metabolic reprogramming in cancer cells

DNA methylation, histone modification, and chromatin remodeling are major epigenetic modifications and have been extensively studied thus far. Noncoding RNAs (ncRNAs) do not encode proteins in the genome and mainly comprise miRNAs, lncRNAs, and circRNAs. NcRNAs account for approximately 80% of the human genome, which contains millions of noncoding regulatory regions. Recently, am increasing number of studies have reported that ncRNAs play important roles in gene expression, epigenetic regulation, and metabolic reprogramming in cancer (Table 1)^107^.

**Table 1:** The regulatory role of noncoding RNAs in cancer cells.

	Noncoding RNAs	Roles in cancer	Cancer	Reference
MiRNA	MiR-431-5p	Inhibits tumor progression	PDAC	^108^
	MiR-601	Promotes cell proliferation by inhibiting the expression of TINP1	GBM	^109^
	MiR-30d	Targets the transcription factor RUNX1 and inhibits the transcriptional activation of the RUNX1-induced Warburg effect	PDAC	^113^
	MiR-21-3p	Promotes cell growth and metastasis	Liver cancer	^114^
	MiR-378a	Inhibits PCa cell proliferation	Prostate cancer	^115^
	MiR-612	Suppresses HCC cell invasion and migration	HCC	^116^
	MiR-4646-5p	Promotes metastasis	GC	^117^
LncRNA	LncRNA H19	Promotes the growth of oral tumors	Oral cancer	^119^
	LncRNA ENO1-IT1	Promotes glycolysis and tumorigenesis	CRC	^120^
	LncRNA NRCP	Promotes cancer cell growth	Ovarian cancer	^121^
	LINC00842	Leads to a metabolic switch from mitochondrial oxidative catabolic processes to fatty acid synthesis, enhancing the malignant phenotype of PDAC cells	PDAC	^122^
	LncRNA ropm	Regulates the properties of breast cancer stem cells (BCSCs) by mediating lipid metabolism	Breast cancer	^123^
		Promotes the survival of glucose-deficient hepatoma cells by activating the serine synthesis pathway	HHC	^124^
	LINC00467	Promotes cell proliferation	CRC	^125^
	Lnc-NA	Regulates the expression of the nuclear receptor NR4A1 and then increases caspase signaling pathway activation, promoting apoptosis in endometrial cell lines	EC	^126^
CircRNA	CircRPN2	Increases the expression of FOXO1 to suppress glucose metabolism and tumor progression	HCC	^130^
	CircMAT2B	Promotes glycolysis through the activation of circMAT2B/miR-338-3p/PKM2 axis to promote HCC progression under hypoxic conditions	HCC	^131^
	CircEZH2	Promotes tumorigenesis and metastasis by sponging miR-217-5p and establishing a new feedback loop consisting of FUS/circEZH2/KLF5	Breast cancer	^132^
	CircECE1	Interacts with c-Myc to prevent POZ-mediated c-Myc ubiquitination and degradation	OS	^133^
	CircErbin	Promotes angiogenesis by increasing the expression of HIF-1α	CRC	^135^
	CircRNF13	Inhibits proliferation and metastasis	NPC	^136^
	CircFNDC3B	Inhibits tumor progression by encoding the protein CircFNDC3B-218aa	Colon cancer	^138^

### MiRNAs regulate metabolic reprogramming in cancer cells

MiRNAs constitute a class of regulatory transcripts that are approximately 19–22 nucleotides in length. MiRNA can be cancer suppressors, such as mir-431-5p, inhibit tumor progression in pancreatic ductal adenocarcinoma cells^108^. MiRNAs can also be oncogenes; for example, miR-601 promotes the proliferation of human glioblastoma cells by inhibiting the expression of TINP1^109^.

Studies have reported that miRNAs are involved in cancerogenesis through various regulatory mechanisms. MiRNAs are key regulators in endoplasmic reticulum stress-related diseases, including cancer. Factors involved in the unfolded protein response (UPR), ER-related degradation (ERAD) and cell death caused by endoplasmic reticulum stress are targeted and regulated by various miRNAs^110^. MiRNAs interact with endothelial cells during tumor progression, promoting or inhibiting tumor angiogenesis or lymphangiogenesis. MiR-9 and miR-494 are usually highly expressed in tumors and induce the expression of angiogenesis-related genes in endothelial cells, while the levels of miR-200 and miR-128, which inhibit angiogenesis and lymphangiogenesis, are frequently reduced^111^. MicroRNAs are mostly involved in regulating all aspects of immune cell development and differentiation. Studies have shown that miRNAs are involved in maintaining lymphocyte tolerance. Moreover, dysregulation of microRNAs contributes to the development of autoimmune diseases and lymphomas^112^. MiRNAs regulate tumor development by regulating metabolic reprogramming in cancer cells. MiR-30d/RUNX1 regulates aerobic glycolysis in pancreatic ductal adenocarcinoma (PDAC) cells. MiR-30d has been recently identified as a target for YTHDC1 m6A modification. YTHDC1-mediated m6A promoted mRNA stability and regulated the biogenesis of mature miR-30d. MiR-30d targets RUNX1, a transcription factor that binds to the promoters of the SLC2A1 and HK1 genes, to regulate the expression of SLC2A1 and HK1, thereby inhibiting the transcriptional activation of the RUNX1-induced Warburg effect and suppressing pancreatic tumorigenesis^113^. LIX1-like protein upregulates miR-21-3p expression and promotes hepatocellular carcinoma growth and metastasis by inhibiting fructose-1,6-bisphosphatase (FBP1)^114^. MiR-378a inhibits prostate (PCa) cell proliferation by inhibiting glucose metabolism that is mediated by GLUT1^115^. Ectopic expression of miR-612 partially decreased HADHA levels, reduced invasive pseudopodia formation through HADHA-mediated changes in cell membrane cholesterol levels, and inhibited the metastasis and invasion of HCC cells via its effect on lipid reprogramming^116^. MiR-4646-5p regulates the secretion of ABHD16A and the lysophospholipid metabolites to promote gastric cancer cell metastasis^117^. In addition, miRNAs generated by tumor cells modulate fibroblast protein synthesis and amino acid-induced mTORC1 activation^118^.

### LncRNAs regulate metabolic reprogramming in cancer cells

LncRNAs are RNA transcripts that carry more than 200 nucleotides and show no or limited protein-coding potential. Recently, many studies have revealed that lncRNAs exhibit complex regulatory roles in metabolic reprogramming in cancer cells and that these effects are mediated through various mechanisms.

LncRNAs affect the glucose metabolism in cancer cells through a wide range of mechanisms. The lncRNA H19/miR-675-5p/PFKFB3 signaling pathway promotes oral cancer development by reprogramming glycolysis in cancer-associated fibroblasts (CAFs)^119^. The relationship between the gut microbiota and glucose metabolism is also mediated via lncRNAs. In patients, *Fusarium nucleatum* (F.) abundance is associated with increased glucose metabolism. *F. nucleatum* increases the binding effectiveness of the transcription factor SP1 to the lncRNA ENO1-IT1 promoter region, activating transcription. Elevated ENO1-IT directs the histone modification of target genes to promote glycolysis and tumorigenesis^120^. The long noncoding RNA ceruloplasmin (the lncRNA NRCP) promotes the development by modifying the glycolysis of tumor cells. NRCP expression is upregulated in ovarian cancer. Silencing NRCP markedly reduces glycolysis and increase mitochondrial respiration in cancer cells, ultimately leading to an increased apoptosis and reduced proliferation rates of cancer cells^121^. LncRNAs are also engaged in lipid and amino acid metabolism in cancer cells. LINC00842 is upregulated in pancreatic ductal adenocarcinoma (PDAC). pGC-1α is an important transcriptional cofactor that participates in regulating cellular metabolism. By binding to acetylated pGC-1, LINC00842 blocks SIRT1 deacetylation of pGC-1, stabilizing the pGC-1 level. The malignant phenotype of PDAC cells was enhanced by overexpression of LINC00842, which triggered metabolic switching from mitochondrial oxidative catabolic processes to fatty acid production^122^. The lncRNA ropm regulates the characteristics of breast cancer stem cells (BCSCs) by mediating lipid metabolic processes. The XVI phospholipase A2 PLA2G16 is a prominent promoter of Group phospholipid metabolism and free fatty acid synthesis, specifically arachidonic acid. lncROPM binds to the PLA2G16’s 3′ UTR to regulate PLA2G16 expression, which in turn activates PI3K/AKT, WNT/Catenin, and HIPPO/YAP signaling, which maintains BCSC stemness^123^. Lnc01564 promotes the survival of glucose-deficient hepatoma cells by activating the SSP. PHGDH is a rate-limiting enzyme in the SPP. Lnc01564 binds to PHGDH in a competitive manner, weakening the suppression of PHGDH induced by miR-107/103a-3p, leading to an increase in PHGDH expression and promoting hepatocellular carcinogenesis and cancer cell survival^124^. A 94-amino acid micropeptide adenosine triphosphate synthase-associated peptide (ASAP) encoded by LINC00467 has been identified in colorectal cancer. The peptide facilitates the assembly of adenosine triphosphate synthase subunits (ATP5A and ATP5C) by interacting with them to increase their activity levels and mitochondrial oxygen consumption rate, thereby favoring colorectal cancer cell proliferation^125^. In addition, lncRNAs are involved in cell apoptosis. Lnc-NA specifically regulates the expression of the nuclear receptor NR4A1 and increasing caspase apoptosis signaling pathway activation, thereby inhibiting tumor cell proliferation, invasion, and migration and promoting apoptosis in endometrial cell lineages^126^.

### CircRNAs regulate metabolic reprogramming in cancer cells

CircRNAs are among the most extensively studied noncoding RNAs in the past few years. CircRNAs are derived from pre-mRNA reverse splicing and form a closed loop structure without a 5’ end cap or a 3’ end polyA tail. Due to their unique closed loop structure, circRNAs are not degraded by exonucleases and have a long half-life^127^. The main finding thus far is that circRNAs function by interacting with RNA-binding proteins (RBPs), sponging mRNAs or being directly translated into peptides^128^. CircRNAs regulate cancer cell metabolism through regulatory mechanisms that have also been increasingly clarified^129^.

CircRNAs regulate metabolic reprogramming through as competitive endogenous RNAs (ceRNAs). CircRNAs adsorb (sponge) miRNAs to prevent miRNA binding to target genes and thus modulate the expression of miRNA-targeted genes. For example, circRPN2 has been shown to bind to enolase 1 (ENO1), accelerating its degradation. CircRPN2 promotes glycolytic reprogramming via the AKT/mTOR pathway to inhibit hepatocellular carcinoma cell metastasis. CircRPN2 is also an endogenous competitor for miR-183-5p binding, which upregulates forkhead box protein O1 (FOXO1) expression to suppress glucose metabolism and tumor progression^130^. The expression of circMAT2B is upregulated in HCC and is linked to the overall survival of HCC patients. Studies have shown that circMAT2B promotes glycolysis by activating the circMAT2B/miR-338-3p/PKM2 axis to induce HCC progression under hypoxic conditions. CircMAT2B positively regulates the expression level of PKM2 by sponging mir-338-3p. The PKM2 gene encodes a key enzyme in glycolysis^131^. CircEZH2 expression is upregulated in breast cancer (BC) liver metastases and is associated with a poor patient prognosis. Mechanistically, circEZH2 upregulates KLF5 expression by sponging miR-217-5p to promote tumorigenesis and metastasis. Moreover, transcriptional activation of FUS induced by KLF5 promotes circEZH2 reverse splicing. Thus, a novel FUS/CircEZH2-KLF5 feedback loop is established. Ultimately, KLF5 upregulates CXCR4 transcription and contributes to the liver metastasis of BC cells by promoting the epithelial-mesenchymal transition^132^.

CircRNAs affect metabolic reprogramming by regulating the expression of transcription factors. circECE1 is expressed at high levels in osteosarcoma (OS) tissues and cells. Knocking down circECE1 inhibits the proliferation and metastasis of tumor cells in vitro and in vivo. Mechanistically, circECE1 and c-Myc interact to hinder POZ-mediated degradation of ubiquitinated c-Myc. c-Myc represses the transcription of thioredoxin-binding protein (TXNIP), which in turn activates aerobic glycolysis^133^. Circ_0010729 was overexpressed in human umbilical vein endothelial cells (HUVECs). Under hypoxic conditions, HUVECs promote endothelial cell proliferation by upregulating the expression of HIF-1α due to miR-186 sponging^134^. Overexpression of circ-Erbin in colorectal cancer cells significantly increased the angiogenesis rate by upregulating HIF-1α expression. CIRC-Erbin sponges miR-125a-5p and miR-138-5p, targeting eukaryotic translation initiation factor 4E-binding protein 1 (4EBP-1) and accelerating HIF-1α capsid protein translation in colorectal cancer cells^135^.

CircRNAs exert effects on cancer metabolism by regulating metabolism-related transporter and enzyme expression. Notably, circRNF13 expression is low in nasopharyngeal carcinoma clinical tissues and nasopharyngeal carcinoma cells. CircRNF13 inhibits the proliferation and metastasis of nasopharyngeal carcinoma cells in vitro and in vivo. Mechanistically, the binding of circRNF13 to the 3’-untranslated region (3’-UTR) of SUMO2 prolonged the SUMO2 gene half-life. SUMO2 glycosylation and GLUT1 ubiquitination promoted GLUT1 degradation and activated the AMPK–mTOR pathway through glycolytic inhibition, leading to nasopharyngeal carcinoma cell proliferation and metastasis^136^. CircACC1 is derived from ACC1 pre-mRNA and is critical to the cellular response to metabolic stress. Under serum deprivation conditions, the transcription factor c-Jun upregulates the expression of circACC1, which destabilizes and promotes AMPK enzyme activity, thereby promoting fatty acid oxidation and glycolysis^137^.

CircRNAs play important roles when they are translated into proteins. The circFNDC3B-encoded protein regulates Snail expression to suppress colon cancer progression. Studies have indicated that circFNDC3B encodes proteins. Through functional experiments, circFNDC3B-218aa was shown to promote cell proliferation, invasion, migration, and glucose metabolism^138^.

How these epigenetic processes are coordinated remains unclear, despite the abundant research being reported. NcRNAs contribute to tumor development through their epigenetic roles in metabolic reprogramming. The functions of ncRNAs in regulating metabolic reprogramming are much more complex and deserve further exploration.

## Epigenetic modification in metabolic reprogramming and cancer therapy

In contrast to genetic mutations, abnormal changes in epigenetic signatures are usually reversible. Leveraging the reversibility of epigenetic modification to normalize abnormal epigenetic modification is a strategy for tumor treatment. For example, DNA methyltransferase inhibitors (DNMTis) and histone deacetylase inhibitors (HDACis) can be used to treat cancers because they change the patterns of DNA methylation and histone modification. DNMT3a catalyzes elevated DNA methylation of gene promoters in prostate cancer (PCa). Polo-like kinase 1 is involved in multiple aspects of the cell cycle. The combined application of Plk1 inhibition and the DNMT3a inhibitor 5-aza-2’-deoxycytidine (5-aza) is a novel and efficient treatment of prostate cancer^139^. KA2507 has been validated as a selective HDAC6 inhibitor. Apostolia M Tsimberidou et al.^140^ conducted the first preclinical model and human evaluations of KA2507, and the results showed its antitumor efficacy and immunomodulatory effect. To date, a variety of DNMT inhibitors and HDAC inhibitors, such as azacitidine, decitabine, vorinostat, belinostat, and romidepsin, have been used to treat certain diseases, such as AML and myelodysplastic syndrome, increasing the choice for cancer patients, especially for those with hematological tumors^141^.

The immune system plays a major antitumor role Cancer cells undergoing metabolism reprogramming interact with immune cells to disrupt normal immune function^142^. Epigenetic mechanisms are crucial for the activation, differentiation, and effector functions of immune cells. Some important immune-related genes, such as granzyme B, interferon-γ, IL-2, IL-12, FoxP3, and STING, and immune checkpoint molecules (such as PD-1, CTLA-4, TIM-3, LAG-3, and TIGIT) can all be regulated in immune and cancer cells through epigenetic mechanisms. Small-molecule inhibitors of epigenetic regulators are expected to strengthen antitumor immune responses^141,143^. In mouse melanomas that are resistant to checkpoint inhibition, for instance, histone demethylase LSD1 knockdown increased tumor immunogenicity and T-cell infiltration, leading to a considerable response to anti-PD-1 therapy. LSD1 combined with anti-PD-1 immunotherapy may be effective against tumors^144^. Researchers found that using DNMTis combined with HDACis to treat NSCLC mice reversed tumor cell immune escape and promoted an increased response of NSCLC cells to immunotherapy^145^. Recently, chimeric antigen receptor (CAR) T-cell therapy has shown great success against cancer. However, several barriers have limited the efficacy of CAR T cells in cancer treatments. These challenges include the actions of immunosuppressive cells and molecules in the TME, inferior durability of CAR T cell expansion in vitro, and barriers to CAR T-cell infiltration and transport to tumor locations. Compared with T cells from healthy people, T cells from individuals with cancer have shown dysregulated epigenetic modification. Several barriers to CAR T-cell therapy may be overcome through epigenetic remodeling that increases T-cell endurance and viability, reduces their depletion, increases their infiltration to facilitate the acquisition of a memory phenotype^146^.

Furthermore, with the emergence of gene therapy, editing the epigenome has become increasingly important to epigenetic modification-based therapy and is worth further exploration. Epigenetic modification editing therapies require agent binding to target DNA while regulating DNA methylation and mediating histone modifications. Long-term studies of DNA-binding domains have led to the discovery of enzymes capable of writing or erasing epigenetic marks. Precise editing of the epigenome is at the forefront of research. Several companies, such as Chroma Medicine and Tune Therapeutics, are working on epigenome editing, and both companies a developing DNA-binding proteins, inactivated versions of Cas9 (“dead” Cas9, dCas9) or zinc finger structural proteins that target different enzymes. The aim is to create therapies that act on specific gene loci to control gene expression at the genome level^147^. Gersbach’s group is conducting large-scale screening to identify candidate epigenome sequences for editing. They have identified a CRISPR‒Cas9-based acetyltransferase that activates promoters and enhancers in the epigenome, providing a powerful tool for manipulating gene regulation^148^. “Epigenome editing” offers a precise therapeutic pathway for targeting site-specific epigenetic disorders, preventing the effects of untargeted editing. In summary, targeted epigenetic mark are reprogrammed. Various gene-editing technologies, such as zinc finger nucleases (ZFNs), transcription activator-like effector nucleases (TALENs), and short palindromic repeat sequences (CRISPR–CAS9), have transformed the landscape of genome editing by offering extraordinary control and accuracy^149^. Future epigenome-editing therapies are expected to lead to revolutionary breakthrough therapies in cancer.

Alterations in the three-dimensional structure of chromatin show promise for cancer therapy. Almassalha et al.^150^ combined simulation, systematic modeling and in vitro experiments to establish a physically regulated framework for regulating genomic information based on heterogeneous chromatin-packing density. CBL0137 is an anticancer compound that restores the function of the tumor suppressors TP53 and RB. Furthermore, the combined use of CBL0137 and panobinostat markedly prolonged the viability of mice transplanted with diffuse intrinsic pontine glioma (DIPG) cells, offering a promising treatment strategy for DIPG^151^.

## Conclusions

In the past two decades, epigenetics in cancer has been greatly advanced, and numerous studies have suggested that epigenetic modifications exert an important regulatory effect on metabolic reprogramming in tumor cells. Similarly, studies have shown that the epigenome is sensitive to the metabolic state of cells and that metabolites play critical roles in the epigenetic reprogramming of oncogenic pathways. Metabolites produced via the glycolytic cycle and in oxidative phosphorylation and other pathways are cofactors or substrates for enzymatic reactions involved in epigenetic modifications and transcriptional regulation, such as S-adenosylmethionine (SAM) and methylation, acetyl coenzyme A and acetylation, and ATP and phosphorylation^18^. Metabolic reprogramming can influence the activity of epigenetic chromatin regulators. Metabolites can function as competitive inhibitors of chromatin modifications^152^. Therefore, the effects of altered metabolism in cancer cells on epigenetic modifications cannot be ignored. Metabolic reprogramming and epigenetic modifications in cancer cells interact in a bidirectional manner. These interactions may contribute to tumorigenesis.

Metabolic variations and aberrant epigenetic regulation are common in many cancers. They are promising targets for anticancer therapy. The reversibility of epigenetic modifications provides opportunities to correct their abnormal modifications. Therefore, using epigenome-targeted drugs to promote the normalization of metabolic reprogramming in cancer cells will become an important strategy for cancer treatment. Although the field has made great progress in recent years, some new problems have emerged, such as poor drug specificity and toxic side effects. Therefore, the development of targeted strategies for specific genes has important scientific and clinical significance.

The EZH2 inhibitor tazemetostat has been approved for EZH2-mutant lymphoma and INI1-deficient soft tissue sarcoma, signaling is a major breakthrough in epigenetics-based medicine. An innovative therapy for epithelioid sarcoma indicates that EZH2 inhibitors show very promising prospects for the treatment of solid tumors that have been difficult to achieve by previously developed epigenetics-based drugs. These major breakthroughs continue to increase the research and development of epigenetic drugs. In addition, the mechanisms by which different epigenetic modifications work together in the same cell are intriguing areas of research. Studies have shown interactions between different modifications^153^. For example, interactions between m6A RNA methylation modifications and other epigenetic marks are involved in tumor progression^154^. Bidirectional regulation between DNA methylation marks and chromatin triggers the methylation or demethylation of DNA^155^. In the future, considerable effort will be needed to characterize the interactions of these epigenetic marks with metabolic reprogramming factors in cancer cells. Exploring potential remedies to correct aberrant epigenetic remodeling and establish metabolic balance in cancer cells show potential for cancer treatments.

## References

[1] The cause of cancer. *JAMA* **325**, 311 (2021). 10.1001/jama.2020.17762 33464327

[2] Martinez-Reyes I, Chandel NS (2021). Cancer metabolism: looking forward. Nat. Rev. Cancer.

[3] Li Z, Zhang H (2016). Reprogramming of glucose, fatty acid and amino acid metabolism for cancer progression. Cell Mol. Life Sci..

[4] Callao V, Montoya E (1961). Toxohormone-like factor from microorganisms with impaired respiration. Science.

[5] Pavlova NN, Thompson CB (2016). The emerging hallmarks of cancer metabolism. Cell Metab..

[6] Hanahan D (2022). Hallmarks of cancer: new dimensions. Cancer Discov..

[7] Waddington CH (1968). Towards a theoretical biology. Nature.

[8] Wu C, Morris JR (2001). Genes, genetics, and epigenetics: a correspondence. Science.

[9] Zhang L, Lu Q, Chang C (2020). Epigenetics in health and disease. Adv. Exp. Med. Biol..

[10] Kaliman P (2019). Epigenetics and meditation. Curr. Opin. Psychol..

[11] Papait R, Serio S, Condorelli G (2020). Role of the epigenome in heart failure. Physiol. Rev..

[12] Baylin, S. B. & Jones, P. A. Epigenetic determinants of cancer. *Cold Spring Harb. Perspect. Biol* **8**, 10.1101/cshperspect.a019505 (2016). 10.1101/cshperspect.a019505 PMC5008069 27194046

[13] Biswas S, Rao CM (2017). Epigenetics in cancer: fundamentals and beyond. Pharmacol. Ther..

[14] Flavahan WA, Gaskell E, Bernstein BE (2017). Epigenetic plasticity and the hallmarks of cancer. Science.

[15] Jones PA, Issa JP, Baylin S (2016). Targeting the cancer epigenome for therapy. Nat. Rev. Genet..

[16] Nepali K, Liou JP (2021). Recent developments in epigenetic cancer therapeutics: clinical advancement and emerging trends. J. Biomed. Sci..

[17] Chen W (2019). Disparities by province, age, and sex in site-specific cancer burden attributable to 23 potentially modifiable risk factors in China: a comparative risk assessment. Lancet Glob. Health.

[18] Sun, L., Zhang, H. & Gao, P. Metabolic reprogramming and epigenetic modifications on the path to cancer. *Protein Cell*10.1007/s13238-021-00846-7 (2021). 10.1007/s13238-021-00846-7 PMC9243210 34050894

[19] Thakur C, Chen F (2019). Connections between metabolism and epigenetics in cancers. Semin. Cancer Biol..

[20] Warburg O (1956). On the origin of cancer cells. Science.

[21] Vaupel P, Multhoff G (2021). The Warburg effect: historical dogma versus current rationale. Adv. Exp. Med. Biol..

[22] Sun L (2015). cMyc-mediated activation of serine biosynthesis pathway is critical for cancer progression under nutrient deprivation conditions. Cell Res..

[23] Kierans SJ, Taylor CT (2021). Regulation of glycolysis by the hypoxia-inducible factor (HIF): implications for cellular physiology. J. Physiol..

[24] Al Tameemi W, Dale TP, Al-Jumaily RMK, Forsyth NR (2019). Hypoxia-modified cancer cell metabolism. Front. Cell Dev. Biol..

[25] Nagao, A., Kobayashi, M., Koyasu, S., Chow, C. C. T. & Harada, H. HIF-1-dependent reprogramming of glucose metabolic pathway of cancer cells and its therapeutic significance. *Int. J. Mol. Sci.* **20**, 10.3390/ijms20020238 (2019). 10.3390/ijms20020238 PMC6359724 30634433

[26] Bao X (2021). The crosstalk between HIFs and mitochondrial dysfunctions in cancer development. Cell Death Dis..

[27] Vander Heiden MG, Cantley LC, Thompson CB (2009). Understanding the Warburg effect: the metabolic requirements of cell proliferation. Science.

[28] Certo M, Tsai CH, Pucino V, Ho PC, Mauro C (2021). Lactate modulation of immune responses in inflammatory versus tumour microenvironments. Nat. Rev. Immunol..

[29] Deng F (2019). Tumor-secreted dickkopf2 accelerates aerobic glycolysis and promotes angiogenesis in colorectal cancer. Theranostics.

[30] Bal C, Chakraborty D, Khan D (2022). Positron emission tomography/computed tomography in thyroid cancer. PET Clin..

[31] Guglielmi R, Andreisek G, Halpern BS (2022). ^18^F FDG imaging - response criteria in tumors. Eur. J. Radiol..

[32] Baier, D. et al. The anticancer ruthenium compound BOLD-100 targets glycolysis and generates a metabolic vulnerability towards glucose deprivation. *Pharmaceutics* **14**, 10.3390/pharmaceutics14020238 (2022). 10.3390/pharmaceutics14020238 PMC8875291 35213972

[33] Snaebjornsson MT, Janaki-Raman S, Schulze A (2020). Greasing the wheels of the cancer machine: the role of lipid metabolism in cancer. Cell Metab..

[34] Bian, X. et al. Lipid metabolism and cancer. *J. Exp. Med.* **218**, 10.1084/jem.20201606 (2021). 10.1084/jem.20201606 PMC7754673 33601415

[35] Gu L (2021). The IKKbeta-USP30-ACLY axis controls lipogenesis and tumorigenesis. Hepatology.

[36] Fhu, C. W. & Ali, A. Fatty acid synthase: an emerging target in cancer. *Molecules* **25**, 10.3390/molecules25173935 (2020). 10.3390/molecules25173935 PMC7504791 32872164

[37] Oatman, N. et al. Mechanisms of stearoyl CoA desaturase inhibitor sensitivity and acquired resistance in cancer. *Sci. Adv.* **7**, 10.1126/sciadv.abd7459 (2021). 10.1126/sciadv.abd7459 PMC7875532 33568479

[38] Ruiz de Gauna, M. et al. Cholangiocarcinoma progression depends on the uptake and metabolization of extracellular lipids. *Hepatology*, 10.1002/hep.32344 (2022). 10.1002/hep.32344 PMC9790564 35030285

[39] Munir R, Lisec J, Swinnen JV, Zaidi N (2022). Too complex to fail? Targeting fatty acid metabolism for cancer therapy. Prog. Lipid Res..

[40] Liu W (2021). Dysregulated cholesterol homeostasis results in resistance to ferroptosis increasing tumorigenicity and metastasis in cancer. Nat. Commun..

[41] King RJ, Singh PK, Mehla K (2022). The cholesterol pathway: impact on immunity and cancer. Trends Immunol..

[42] Lim SA (2021). Lipid signalling enforces functional specialization of Treg cells in tumours. Nature.

[43] Lieu EL, Nguyen T, Rhyne S, Kim J (2020). Amino acids in cancer. Exp. Mol. Med..

[44] Yoo HC, Yu YC, Sung Y, Han JM (2020). Glutamine reliance in cell metabolism. Exp. Mol. Med..

[45] Wei Z, Liu X, Cheng C, Yu W, Yi P (2020). Metabolism of amino acids in cancer. Front. Cell Dev. Biol..

[46] Baksh SC (2020). Extracellular serine controls epidermal stem cell fate and tumour initiation. Nat. Cell Biol..

[47] Muthusamy T (2020). Serine restriction alters sphingolipid diversity to constrain tumour growth. Nature.

[48] Tajan M (2021). Serine synthesis pathway inhibition cooperates with dietary serine and glycine limitation for cancer therapy. Nat. Commun..

[49] Hayes JD, Dinkova-Kostova AT, Tew KD (2020). Oxidative stress in cancer. Cancer Cell.

[50] Ying M (2021). Lactate and glutamine support NADPH generation in cancer cells under glucose deprived conditions. Redox Biol..

[51] Ballestar E, Sawalha AH, Lu Q (2020). Clinical value of DNA methylation markers in autoimmune rheumatic diseases. Nat. Rev. Rheumatol..

[52] Navas-Acien A (2021). Blood DNA methylation and incident coronary heart disease: evidence from the strong heart study. JAMA Cardiol.

[53] Nishiyama A, Nakanishi M (2021). Navigating the DNA methylation landscape of cancer. Trends Genet..

[54] Xu, Q. et al. Loss of TET reprograms Wnt signaling through impaired demethylation to promote lung cancer development. *Proc. Natl Acad. Sci. USA* **119**, 10.1073/pnas.2107599119 (2022). 10.1073/pnas.2107599119 PMC8832965 35110400

[55] Wong KK (2021). DNMT1: A key drug target in triple-negative breast cancer. Semin Cancer Biol..

[56] Wu D (2018). Glucose-regulated phosphorylation of TET2 by AMPK reveals a pathway linking diabetes to cancer. Nature.

[57] Pulikkottil AJ (2022). TET3 promotes AML growth and epigenetically regulates glucose metabolism and leukemic stem cell associated pathways. Leukemia.

[58] Le X (2020). DNA methylation downregulated ZDHHC1 suppresses tumor growth by altering cellular metabolism and inducing oxidative/ER stress-mediated apoptosis and pyroptosis. Theranostics.

[59] Feng J (2019). Hypermethylated gene ANKDD1A is a candidate tumor suppressor that interacts with FIH1 and decreases HIF1alpha stability to inhibit cell autophagy in the glioblastoma multiforme hypoxia microenvironment. Oncogene.

[60] Zhong X (2021). Downregulation of SLC27A6 by DNA hypermethylation promotes proliferation but suppresses metastasis of nasopharyngeal carcinoma through modulating lipid metabolism. Front. Oncol..

[61] Chen Y (2020). The role of histone methylation in the development of digestive cancers: a potential direction for cancer management. Signal Transduct. Target. Ther..

[62] Tran TQ, Lowman XH, Kong M (2017). Molecular pathways: metabolic control of histone methylation and gene expression in cancer. Clin. Cancer Res..

[63] Yuan G (2021). Elevated NSD3 histone methylation activity drives squamous cell lung cancer. Nature.

[64] Koutsioumpa M (2019). Lysine methyltransferase 2D regulates pancreatic carcinogenesis through metabolic reprogramming. Gut.

[65] Alam H (2020). KMT2D deficiency impairs super-enhancers to confer a glycolytic vulnerability in lung cancer. Cancer Cell.

[66] Maitituoheti M (2020). Enhancer reprogramming confers dependence on glycolysis and IGF signaling in KMT2D mutant melanoma. Cell Rep..

[67] Wu J, Chai H, Xu X, Yu J, Gu Y (2020). Histone methyltransferase SETD1A interacts with HIF1alpha to enhance glycolysis and promote cancer progression in gastric cancer. Mol. Oncol..

[68] Wan W (2017). Histone demethylase JMJD1A promotes urinary bladder cancer progression by enhancing glycolysis through coactivation of hypoxia inducible factor 1alpha. Oncogene.

[69] Zhang ZG (2019). KDM5B promotes breast cancer cell proliferation and migration via AMPK-mediated lipid metabolism reprogramming. Exp. Cell Res..

[70] Matsushita H (2006). In vivo analysis of the role of aberrant histone deacetylase recruitment and RAR alpha blockade in the pathogenesis of acute promyelocytic leukemia. J. Exp. Med..

[71] An X (2020). Histone deacetylase inhibitor trichostatin a suppresses cell proliferation and induces apoptosis by regulating the PI3K/AKT signalling pathway in gastric cancer cells. Anticancer Agents Med. Chem..

[72] Cai LY (2021). Targeting p300/CBP attenuates hepatocellular carcinoma progression through epigenetic regulation of metabolism. Cancer Res..

[73] Jiang X (2022). BPIFB1 inhibits vasculogenic mimicry via downregulation of GLUT1-mediated H3K27 acetylation in nasopharyngeal carcinoma. Oncogene.

[74] Chen CL (2021). Arginine is an epigenetic regulator targeting TEAD4 to modulate OXPHOS in prostate cancer cells. Nat. Commun..

[75] Rabinowitz JD, Enerback S (2020). Lactate: the ugly duckling of energy metabolism. Nat. Metab..

[76] Brown TP, Ganapathy V (2020). Lactate/GPR81 signaling and proton motive force in cancer: role in angiogenesis, immune escape, nutrition, and Warburg phenomenon. Pharmacol. Ther..

[77] Daw CC (2020). Lactate elicits ER-mitochondrial Mg(2+) dynamics to integrate. Cellular Metabolism. Cell.

[78] Lundo K, Trauelsen M, Pedersen SF, Schwartz TW (2020). Why Warburg works: lactate controls immune evasion through GPR81. Cell Metab.

[79] Zhang D (2019). Metabolic regulation of gene expression by histone lactylation. Nature.

[80] Yu J (2021). Histone lactylation drives oncogenesis by facilitating m(6)A reader protein YTHDF2 expression in ocular melanoma. Genome Biol..

[81] Xiong J (2022). Lactylation-driven METTL3-mediated RNA m(6)A modification promotes immunosuppression of tumor-infiltrating myeloid cells. Mol. Cell.

[82] Jiang J (2021). Lactate modulates cellular metabolism through histone lactylation-mediated gene expression in non-small cell lung cancer. Front. Oncol..

[83] Vinasco K, Mitchell HM, Kaakoush NO, Castano-Rodriguez N (2019). Microbial carcinogenesis: lactic acid bacteria in gastric cancer. Biochim. Biophys. Acta Rev. Cancer.

[84] Bhagat, T. D. et al. Lactate-mediated epigenetic reprogramming regulates formation of human pancreatic cancer-associated fibroblasts. *Elife* **8**, 10.7554/eLife.50663 (2019). 10.7554/eLife.50663 PMC6874475 31663852

[85] Yang, Z. et al. Lactylome analysis suggests lactylation-dependent mechanisms of metabolic adaptation in hepatocellular carcinoma. *Nat. Metab.*10.1038/s42255-022-00710-w (2023). 10.1038/s42255-022-00710-w 36593272

[86] Kebede AF, Schneider R, Daujat S (2015). Novel types and sites of histone modifications emerge as players in the transcriptional regulation contest. FEBS J..

[87] Liu H (2021). Rab26 suppresses migration and invasion of breast cancer cells through mediating autophagic degradation of phosphorylated Src. Cell Death Dis..

[88] Zhu D, Zhang Y, Wang S (2021). Histone citrullination: a new target for tumors. Mol. Cancer.

[89] Thomas, D., Rathinavel, A. K. & Radhakrishnan, P. Altered glycosylation in cancer: a promising target for biomarkers and therapeutics. *Biochim. Biophys. Acta (BBA) Rev. Cancer* **1875**, 10.1016/j.bbcan.2020.188464 (2021). 10.1016/j.bbcan.2020.188464 PMC7855613 33157161

[90] Sun L (2020). Targeting glycosylated PD-1 induces potent antitumor immunity. Cancer Res..

[91] Dang F, Nie L, Wei W (2021). Ubiquitin signaling in cell cycle control and tumorigenesis. Cell Death Differ..

[92] Li X (2021). CUL3 (cullin 3)-mediated ubiquitination and degradation of BECN1 (beclin 1) inhibit autophagy and promote tumor progression. Autophagy.

[93] Liu J (2020). UFMylation maintains tumour suppressor p53 stability by antagonizing its ubiquitination. Nat. Cell Biol..

[94] Clapier CR, Cairns BR (2009). The biology of chromatin remodeling complexes. Annu. Rev. Biochem..

[95] Kadoch, C. Diverse compositions and functions of chromatin remodeling machines in cancer. *Sci. Transl. Med.* **11**, 10.1126/scitranslmed.aay1018 (2019). 10.1126/scitranslmed.aay1018 PMC6755912 31316005

[96] Mittal P, Roberts CWM (2020). The SWI/SNF complex in cancer - biology, biomarkers and therapy. Nat. Rev. Clin. Oncol..

[97] Saqcena M (2021). SWI/SNF complex mutations promote thyroid tumor progression and insensitivity to redifferentiation therapies. Cancer Discov..

[98] Ding H (2021). Attenuated expression of SNF5 facilitates progression of bladder cancer via STAT3 activation. Cancer Cell Int..

[99] Lin H, Wong RP, Martinka M, Li G (2009). Loss of SNF5 expression correlates with poor patient survival in melanoma. Clin. Cancer Res..

[100] Wu S (2021). Targeting glutamine dependence through GLS1 inhibition suppresses ARID1A-inactivated clear cell ovarian carcinoma. Nat. Cancer.

[101] Ogiwara H (2019). Targeting the vulnerability of glutathione metabolism in ARID1A-deficient cancers. Cancer Cell.

[102] Wu Q (2016). The BRG1 chromatin remodeling enzyme links cancer cell metabolism and proliferation. Oncotarget.

[103] Li Y (2021). The emerging role of ISWI chromatin remodeling complexes in cancer. J. Exp. Clin. Cancer Res..

[104] Zikmund, T. et al. Loss of ISWI ATPase SMARCA5 (SNF2H) in acute myeloid leukemia cells inhibits proliferation and chromatid cohesion. *Int. J. Mol. Sci.* **21**, 10.3390/ijms21062073 (2020). 10.3390/ijms21062073 PMC7139293 32197313

[105] Zhou B (2016). INO80 governs superenhancer-mediated oncogenic transcription and tumor growth in melanoma. Genes Dev..

[106] Prendergast L (2020). Resolution of R-loops by INO80 promotes DNA replication and maintains cancer cell proliferation and viability. Nat. Commun..

[107] Zhou B (2021). Translation of noncoding RNAs and cancer. Cancer Lett..

[108] Haugen, O. P., Khuu, C., Weidemann, H. M., Paaske Utheim, T. & Hildegard Bergersen, L. Transcriptomic and functional studies reveal miR-431-5p as a tumour suppressor in pancreatic ductal adenocarcinoma cells. *Gene* **822**, 146346 (2022). 10.1016/j.gene.2022.146346 35182679

[109] Chen L, Zhu S, Wang H, Pang X, Wang X (2022). MiR-601 promotes cell proliferation of human glioblastoma cells by suppressing TINP1 expression. Altern. Ther. Health Med..

[110] Kim T, Croce CM (2021). MicroRNA and ER stress in cancer. Semin. Cancer Biol..

[111] Orso F (2020). Role of miRNAs in tumor and endothelial cell interactions during tumor progression. Semin. Cancer Biol..

[112] Xiao C, Nemazee D, Gonzalez-Martin A (2020). MicroRNA control of B cell tolerance, autoimmunity and cancer. Semin. Cancer Biol..

[113] Hou Y (2021). YTHDC1-mediated augmentation of miR-30d in repressing pancreatic tumorigenesis via attenuation of RUNX1-induced transcriptional activation of Warburg effect. Cell Death Differ..

[114] Zou J (2021). LIX1-like protein promotes liver cancer progression via miR-21-3p-mediated inhibition of fructose-1,6-bisphosphatase. Acta Pharm. Sin. B.

[115] Cannistraci A (2022). MiR-378a inhibits glucose metabolism by suppressing GLUT1 in prostate cancer. Oncogene.

[116] Liu Y (2020). MiR-612 regulates invadopodia of hepatocellular carcinoma by HADHA-mediated lipid reprogramming. J. Hematol. Oncol..

[117] Yang L (2021). Mirtronic miR-4646-5p promotes gastric cancer metastasis by regulating ABHD16A and metabolite lysophosphatidylserines. Cell Death Differ..

[118] Fong MY (2021). Cancer-secreted miRNAs regulate amino-acid-induced mTORC1 signaling and fibroblast protein synthesis. EMBO Rep..

[119] Yang J (2021). Glycolysis reprogramming in cancer-associated fibroblasts promotes the growth of oral cancer through the lncRNA H19/miR-675-5p/PFKFB3 signaling pathway. Int. J. Oral Sci..

[120] Hong J (2021). F. nucleatum targets lncRNA ENO1-IT1 to promote glycolysis and oncogenesis in colorectal cancer. Gut.

[121] Rupaimoole R (2015). Long noncoding RNA ceruloplasmin promotes cancer growth by altering glycolysis. Cell Rep..

[122] Huang X (2021). LINC00842 inactivates transcription co-regulator PGC-1alpha to promote pancreatic cancer malignancy through metabolic remodelling. Nat. Commun..

[123] Liu S (2021). A novel lncRNA ROPM-mediated lipid metabolism governs breast cancer stem cell properties. J. Hematol. Oncol..

[124] Zhang G (2021). Energy stress-induced linc01564 activates the serine synthesis pathway and facilitates hepatocellular carcinogenesis. Oncogene.

[125] Ge, Q. et al. Micropeptide ASAP encoded by LINC00467 promotes colorectal cancer progression by directly modulating ATP synthase activity. *J. Clin. Invest.* **131**, 10.1172/JCI152911 (2021). 10.1172/JCI152911 PMC8592539 34591791

[126] Sun L (2019). Lnc-NA inhibits proliferation and metastasis in endometrioid endometrial carcinoma through regulation of NR4A1. J. Cell Mol. Med..

[127] Kristensen LS (2019). The biogenesis, biology and characterization of circular RNAs. Nat. Rev. Genet..

[128] Pamudurti NR (2017). Translation of CircRNAs. Mol. Cell.

[129] Yu T (2019). CircRNAs in cancer metabolism: a review. J. Hematol. Oncol..

[130] Li J (2022). CircRPN2 inhibits aerobic glycolysis and metastasis in hepatocellular carcinoma. Cancer Res..

[131] Li Q (2019). Circular RNA MAT2B promotes glycolysis and malignancy of hepatocellular carcinoma through the miR-338-3p/PKM2 axis under hypoxic stress. Hepatology.

[132] Liu P (2022). The FUS/circEZH2/KLF5/ feedback loop contributes to CXCR4-induced liver metastasis of breast cancer by enhancing epithelial-mesenchymal transition. Mol. Cancer.

[133] Shen S (2020). CircECE1 activates energy metabolism in osteosarcoma by stabilizing c-Myc. Mol. Cancer.

[134] Dang RY, Liu FL, Li Y (2017). Circular RNA hsa_circ_0010729 regulates vascular endothelial cell proliferation and apoptosis by targeting the miR-186/HIF-1alpha axis. Biochem. Biophys. Res. Commun..

[135] Chen LY (2020). The circular RNA circ-ERBIN promotes growth and metastasis of colorectal cancer by miR-125a-5p and miR-138-5p/4EBP-1 mediated cap-independent HIF-1alpha translation. Mol. Cancer.

[136] Mo Y (2021). Circular RNA circRNF13 inhibits proliferation and metastasis of nasopharyngeal carcinoma via SUMO2. Mol. Cancer.

[137] Li Q (2019). CircACC1 regulates assembly and activation of AMPK complex under metabolic stress. Cell Metab..

[138] Pan Z (2020). A novel protein encoded by circFNDC3B inhibits tumor progression and EMT through regulating Snail in colon cancer. Mol. Cancer.

[139] Zhang Z (2021). Co-targeting Plk1 and DNMT3a in advanced prostate cancer. Adv. Sci..

[140] Tsimberidou AM (2021). Preclinical development and first-in-human study of KA2507, a selective and potent inhibitor of histone deacetylase 6, for patients with refractory solid tumors. Clin. Cancer Res..

[141] Hogg SJ, Beavis PA, Dawson MA, Johnstone RW (2020). Targeting the epigenetic regulation of antitumour immunity. Nat. Rev. Drug Discov..

[142] Xia L (2021). The cancer metabolic reprogramming and immune response. Mol. Cancer.

[143] Dai E (2021). Epigenetic modulation of antitumor immunity for improved cancer immunotherapy. Mol. Cancer.

[144] Sheng W (2018). LSD1 ablation stimulates anti-tumor immunity and enables checkpoint blockade. Cell.

[145] Topper MJ (2017). Epigenetic therapy ties MYC depletion to reversing immune evasion and treating lung cancer. Cell.

[146] Akbari B (2021). Epigenetic strategies to boost CAR T cell therapy. Mol. Ther..

[147] DeFrancesco, L. Chroma Medicine and Tune Therapeutics: two companies take up epigenome editing. *Nat. Biotechnol*. 10.1038/d41587-022-00009-x (2022). 10.1038/d41587-022-00009-x 36058989

[148] Hilton IB (2015). Epigenome editing by a CRISPR-Cas9-based acetyltransferase activates genes from promoters and enhancers. Nat. Biotechnol..

[149] Ansari I, Chaturvedi A, Chitkara D, Singh S (2022). CRISPR/Cas mediated epigenome editing for cancer therapy. Semin. Cancer Biol..

[150] Almassalha LM (2017). Macrogenomic engineering via modulation of the scaling of chromatin packing density. Nat. Biomed. Eng.

[151] Ehteda A (2021). Dual targeting of the epigenome via FACT complex and histone deacetylase is a potent treatment strategy for DIPG. Cell Rep..

[152] Ge T (2022). Crosstalk between metabolic reprogramming and epigenetics in cancer: updates on mechanisms and therapeutic opportunities. Cancer Commun..

[153] Zhang Y, Reinberg D (2001). Transcription regulation by histone methylation: interplay between different covalent modifications of the core histone tails. Genes Dev..

[154] Zhao Y, Chen Y, Jin M, Wang J (2021). The crosstalk between m(6)A RNA methylation and other epigenetic regulators: a novel perspective in epigenetic remodeling. Theranostics.

[155] D’Alessio AC, Szyf M (2006). Epigenetic tete-a-tete: the bilateral relationship between chromatin modifications and DNA methylation. Biochem. Cell Biol..

